# Novel Echocardiography-Derived Left Ventricular Stiffness Index in Low-Flow Versus Normal-Flow Severe Aortic Stenosis with Preserved Left Ventricular Ejection Fraction

**DOI:** 10.1038/s41598-020-65758-8

**Published:** 2020-06-03

**Authors:** Jinghao Nicholas Ngiam, Nicholas WS Chew, Benjamin Yong-Qiang Tan, Hui Wen Sim, William K. F. Kong, Tiong-Cheng Yeo, Shahryar M. Chowdhury, Kian-Keong Poh

**Affiliations:** 1 Department of Medicine, National University Health System, Singapore, Singapore; 2 Department of Cardiology, National University Heart Centre Singapore, National University Health System, Singapore, Singapore; 3 Yong Loo Lin School of Medicine, National University of Singapore, Singapore, Singapore; 4 Department of Pediatric Cardiology, Medical University of South Carolina, Charleston, USA

**Keywords:** Cardiology, Valvular disease

## Abstract

**Background** Paradoxical low-flow (LF) severe aortic stenosis (AS) with preserved left ventricular ejection fraction (LVEF) may have poorer prognosis than normal-flow (NF) AS, though its pathophysiology remained unclear. In particular, LV stiffness has not been compared between LF vs NF. We used a novel echocardiography-derived index of LV stiffness to compare between these groups. Consecutive patients with medically-managed isolated severe AS (aortic valve area < 1 cm^2^) and preserved LVEF (>50%) were studied. Echocardiographic LV stiffness index was measured by a method previously validated against cardiac catheterization. We compared LF (stroke volume index, SVI < 35 ml/m^2^) and NF severe AS. Of the 352 patients, 121 (34%) were LF. Both LF and NF groups had similar demographics, valve areas and indices. Compared to NF, LF severe AS had higher LV stiffness indices (>0.11 ml^−1^ OR 3.067, 95% CI 1.825–5.128, p < 0.001). Increased LV stiffness was associated with concentric remodelling and more severe diastolic dysfunction, especially in LF AS. An LV stiffness index of > 0.11 ml^−1^ was independently associated with increased mortality, after adjusting for age, clinical and echocardiographic parameters (HR 2.283 95% CI 1.318–3.968, p = 0.003). Non-invasive echocardiographic-derived index of LV stiffness may be important in LF AS. Increased LV stiffness was related to LV concentric remodelling and diastolic dysfunction, and associated with poorer clinical outcomes in medically-managed AS.

## Introduction

Significant differences in echocardiographic profiles exist between paradoxical low-flow (LF) and normal-flow (NF) severe aortic stenosis (AS). This may be due to differences in pathophysiological processes in the natural history of LF compared to NF AS. Of note, left ventricular (LV) stiffness has not been evaluated in AS. Conventional measurement of LV stiffness involves using invasive cardiac catheterization and determination of the end-diastolic pressure-volume relationship by pressure-volume loop analysis^1^. This is impractical for routine serial evaluation in AS as it requires invasive cardiac catheterization^2,3^.

AS remains an important disease because of its high prevalence. It affects approximately 5% of patients over 75 years of age, and is associated with reduced survival^4,5^. LF AS (LV stroke volume index (SVI) < 35 mL/m^2^) despite preserved left ventricular ejection fraction (LVEF), termed “paradoxical low-flow”, has been increasingly recognised as a subgroup of severe AS that portends a worse prognosis compared to normal-flow^6^. However, the differential effects of the aortic valve pathology on the LV in LF compared to NF AS, particularly LV stiffness, remains to be elucidated^7–9^.

In addition, several studies have identified predictors of poor prognosis and outcomes in patients with severe AS^10–12^. These studies examined clinical, biochemical, and echocardiographic parameters^13,14^. For example, global longitudinal strain, valvuloarterial impedance, stroke work loss, aortic valve resistance, and systemic arterial compliance have been shown to be important prognostic markers of clinical outcomes in severe NF AS that predicted mortality^15–18^. However, these have not been demonstrated to be useful in paradoxical LF AS^6^.

LV stiffness remained to be evaluated to LF compared with NF AS, and prognostic markers in this subgroup of patients were poorly understood. We thus aimed to evaluate the role of an echocardiography-derived measure of LV stiffness in medically-managed LF versus NF severe AS.

## Methods

We examined the index echocardiographic studies of consecutive patients with severe AS (aortic valve area <1 cm^2^) from 2000–2011 with preserved LVEF (>50%) that were on medical therapy. Patients who had concomitant valvular pathology involving other valves of at least moderate severity, and patients who underwent valve replacement (surgical or transcatheter) were excluded from our study. These patients may be asymptomatic or have either declined valve replacement or were deemed to be medically unfit for the procedures. The patients were then stratified into low-flow (SVI < 35 mL/m^2^) and normal-flow groups. This classification of AS severity was in accordance with the American Society of Echocardiography/European Association of Cardiovascular Imaging guidelines^19,20^. The LV outflow track (OT) diameter was measured at the parasternal long-axis view from inner edge to inner edge. The LVOT velocity was measured with pulsed Doppler from the apical view. The LVOT diameter and LVOT velocity measurements were performed at the same annular level^19^. All echocardiographic measurements were made by independent and certified cardiologists.

Demographic, clinical and echocardiographic parameters were collected. Clinical outcomes in the form of all-cause mortality were collected upon chart review of subsequent follow-up visits.

All echocardiographic parameters were measured in accordance with the guidelines and standards of the American Society of Echocardiography/European Association of Cardiovascular Imaging^21^. Besides conventional echocardiography, tissue Doppler assessment of mitral annular systolic (S’), early (E’) and late (A’) diastolic velocities was performed. Severity of diastolic function was graded according to the American Society of Echocardiography/European Association of Cardiovascular Imaging guidelines^22^. Important prognostic markers of severe AS that were previously identified were also assessed.

LV stiffness was measured by means of a novel echocardiographic index, derived from the following equation^2,3^:

$$LV\,Stiffness\,index=\frac{{\rm{Transmitral}}\,{\rm{E}}:{\rm{lateral}}\,{\rm{mitral}}\,{\rm{annular}}\,{\rm{e}}{\prime} \,}{{\rm{End}}-{\rm{diastolic}}\,{\rm{volume}}}$$

Patients were grouped based on four categories of LV geometry; determined by left ventricular mass index (LVMI) and relative wall thickness (RWT). The cut-off for LVMI was 116 g/m^2^ for males and 104 g/m^2^ for females. The cut-off for RWT was 0.43 for both sexes. If RWT and LVMI were both below the cut-off points, then the patient had normal LV geometry. Patients with high LVMI but RWT < 0.43 were defined as eccentric hypertrophy. Patients with high RWT but LVMI below the cut-off were defined as having concentric remodelling. If both LVMI and RWT were above the cut-offs, then the patient was identified as concentric hypertrophy^24^.

Differences in continuous variables between groups were compared using Student’s t-tests. The association between LV stiffness index and diastolic function grade was assessed using Spearman’s rank correlation. Multivariable logistic regression was used to compare differences between LF versus NF AS. We entered baseline clinical and echocardiographic parameters that were statistically significant on univariate analyses (p < 0.05) into the multivariable model. Parameters that were collinear with SVI and the LV stiffness index were excluded.

The optimized cut-off value for LV stiffness index for predicting all-cause mortality in the entire study population was determined by Youden index using receiver operating characteristic curve analysis. Differences in all-cause mortality between patients with low versus high LV stiffness were assessed using Kaplan-Meier curves and calculating the log-rank test statistic. A multivariate Cox regression model was then constructed to evaluate the effect of increased stiffness in predicting mortality and to adjust for other confounding parameters associated with mortality. We included all baseline clinical and echocardiographic parameters that were statistically significant on univariate analysis (p < 0.05), but excluded parameters that were collinear with the LV stiffness index. All statistical analyses were performed with SPSS for Windows, Version 20.0, SPSS Inc, Chicago, IL. A p-value of less than 0.05 was considered to be statistically significant.

Ethics approval for this study was obtained from National Healthcare Group Domain Specific Review Board (DSRB) prior to the conduct of this study. The study was in compliance with all DSRB requirements based on the Declaration of Helsinki, ethical principles in the Belmont report and the guidelines stipulated by the Bioethics Advisory Committee. No patient identifiers were collected and a waiver for the need for informed consent was obtained from the DSRB.

## Results

Of the 352 patients with severe AS, 121 (34%) were low-flow, while the remaining 231 (66%) were normal-flow. The patients in the LF group were older (73.5 ± 13.8 vs 69.9 ± 13.5 years), but otherwise similar in terms of demographic background (Table 1). There was no significant difference in terms of LVEF and end-systolic wall stress, but end-diastolic volume (70.8 ± 17.7 vs 113.0 ± 26.0 mL) and left ventricular mass index (100.3 ± 31.4 vs 129.8 ± 36.2 g/m^2^) were significantly lower in the LF group (Table 1).

**Table 1:** Patient characteristics and echocardiographic measurements in low-flow (SVI < 35 ml/m^2^) versus normal-flow (SVI ≥ 35 ml/m^2^) severe AS (AVA ≤ 0.1) with preserved LVEF (>50%).

Variables	Low-flow (n = 121)	Normal-flow (n = 231)	Mean Difference/Odds Ratio (95% Confidence Interval)	p-value
Age	73.5 (±13.8)	69.9 (±13.5)	3.6 (0.6–6.6)	0.020
Height (cm)	164.6 (±19.7)	156.0 (±17.2)	8.6 (−3.7–20.8)	0.169
Weight (kg)	60.5 (±12.4)	58.8 (±13.3)	1.6 (−1.2–4.5)	0.268
BSA (m^2^)	1.63 (±0.29)	1.59 (±0.25)	0.03 (−0.01–0.10)	0.136
Hypertension	68.5%	61.9%	1.34 (0.791–1.32)	0.275
Diabetes mellitus	47.8%	35.6%	1.66 (1.01–2.75)	0.047
Hyperlipidaemia	55.4%	41.8%	1.74 (1.05–2.86)	0.030
Atrial fibrillation	11.9%	7.2%	1.41 (0.634–3.16)	0.396
Ischaemic heart disease	34.8%	28.4%	1.34 (0.793–2.29)	0.269
Chronic kidney disease	20.6%	10.8%	2.14 (1.08–4.22)	0.025
**Echocardiographic measurement**				
Left ventricular ejection fraction (%)	65.2 (±7.5)	66.5 (±7.2)	−1.3 (−2.9–0.3)	0.116
End-diastolic volume (ml)	70.8 (±17.7)	113.0 (±26.0)	−42.2 (−47.4 – −37.6)	<0.001
Stroke volume index (ml/m^2^)	28.3 (±5.9)	46.7 (±9.5)	−18.3 (−20.2–16.4)	<0.001
Left ventricular mass index (g/m^2^)	100.3 (±31.4)	129.8 (±36.2)	−29.5 (−37.3 – −21.7)	<0.001
End-systolic wall stress (x 10^3^ dyn/cm^2^)	65.0 (±28.3)	76.3 (±36.2)	−11.3 (−23.0–0.4)	0.058
**Aortic valve indices**				
Aortic valve area (cm^2^)	0.77 (±0.17)	0.78 (±0.17)	−0.01 (−0.05–0.03)	0.633
Transaortic mean pressure gradient (mmHg)	34.1 (±19.8)	36.7 (±20.3)	−2.6 (−7.0–1.9)	0.260
Transaortic peak velocity (cm/s)	367.5 (±94.6)	383.2 (±96.2)	−15.7 (−36.9–5.4)	0.144
**LV diastolic function**				
E (cm/s)	83.8 (±31.5)	89.4 (±30.7)	−5.7 (−12.5–1.2)	0.104
A (cm/s)	89.9 (±42.1)	89.7 (±38.6)	0.2 (−8.6–9.0)	0.965
E/A ratio	2.03 (±1.01)	0.94 (±0.77)	1.1 (−0.8–3.0)	0.264
Deceleration time (ms)	203.1 (±91.4)	211.0 (±83.7)	−7.9 (−27.0–11.2)	0.415
Septal e’	5.39 (±3.65)	5.02 (±3.64)	0.37 (−0.44–1.17)	0.371
Septal E/e’	13.3 (±8.5)	12.4 (±9.2)	0.87 (−1.21–2.95)	0.411
Lateral e’	6.27 (±3.90)	5.70 (4.00)	0.57 (−0.31–1.44)	0.204
Lateral E/e’	10.73 (±6.12)	10.53 (±7.32)	0.20 (−1.43–1.83)	0.809
**LV systolic function**				
Septal S’ (cm/s)	5.50 (±3.27)	4.98 (±3.42)	0.52 (−0.22–1.27)	0.166
Lateral S’ (cm/s)	6.51 (±3.90)	5.86 (±3.86)	0.65 (−0.20–1.51)	0.133
**Prognostic markers in AS**				
Stroke work loss (%)	25.7 (±16.2)	28.1 (±18.0)	−2.4 (−6.2–1.5)	0.223
Systemic arterial compliance (ACU)	0.49 (±0.20)	0.81 (±0.36)	−0.32 (−0.39–0.26)	<0.001
Global LV contractility index, dσ*/dtmax	2.73 (±1.27)	2.41 (±1.00)	0.31 (−0.08–0.72)	0.117
Aortic valve resistance (dyn s /cm^2^)	198.7 (±155.0)	190.7 (±162.5)	8.0 (−27.3–43.3)	0.656
Valvuloarterial impedance (mmHg/ml/m^2^)	5.48 (±4.36)	3.04 (±0.79)	2.44 (1.86–3.02)	<0.001
**LV Stiffness Index**				
Lateral E:e’/EDV (ml^-1^)	0.165 (±0.130)	0.098 (±0.072)	0.066 (0.044–0.089)	<0.001

The values are expressed as mean ± standard deviation.

Patients with LF AS did not differ significantly from those with NF in aortic valve area, transaortic mean pressure gradient or peak velocity. The tissue Doppler indices of systolic and diastolic function were also not significantly different between groups. Conventional prognostic markers of AS severity such as stroke work loss and aortic valve resistance were not significantly different between the LF and NF groups. Systemic arterial compliance was lower and valvuloarterial impedance higher in LF AS compared with NF.

**Figure 1:**
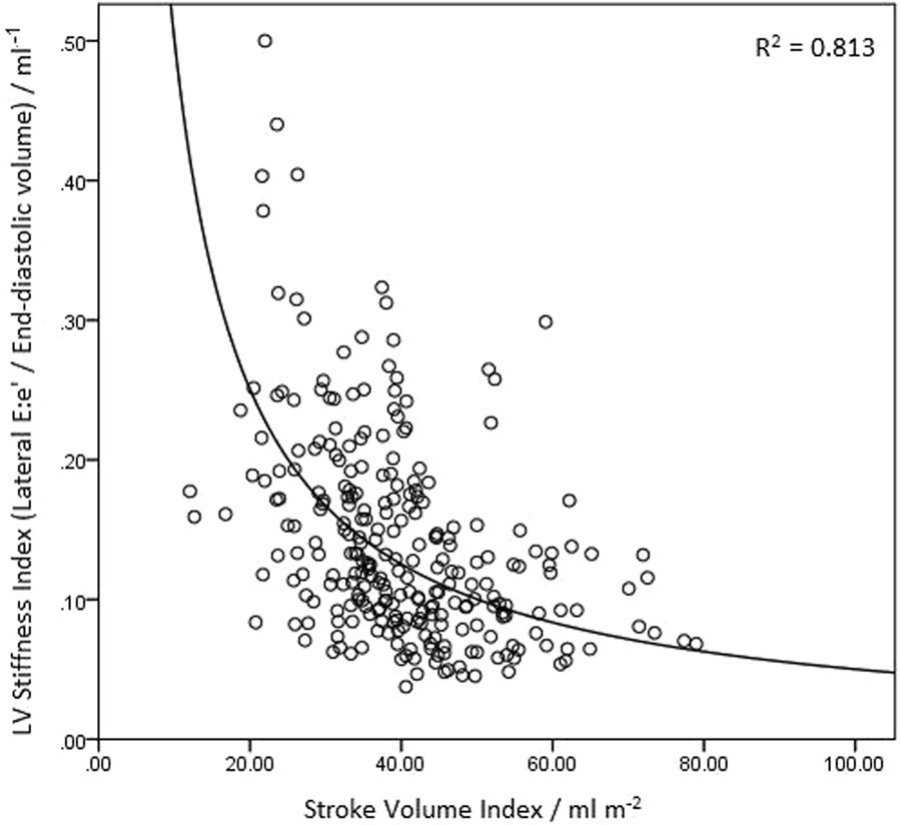
Inverse relationship between LV stiffness and Stroke Volume Index.

There was a strong negative logarithmic correlation between LV stiffness index and SVI (R^2^ = 0.813, Fig. 1). Receiver operating characteristic curve analysis demonstrated an area under the curve of 0.69 (95% CI 0.63–0.75, p < 0.001) and the derived optimised cut-off for LV stiffness index was >0.111 mL^−1^, based on Youden index. After adjusting for the confounding effect of other parameters associated with LF AS, we showed that patients with LF AS had a significantly increased odds of displaying an elevated LV stiffness index (>0.111 mL^−1^) compared to patients with normal-flow AS (OR 3.03, p < 0.001) (Table 2).

**Table 2:** Multivariate regression analysis comparing low-flow (SVI < 35 ml/m^2^) to normal-flow (SVI ≥ 35 ml/m^2^) severe AS (AVA ≤ 1cm^2^) with preserved LVEF (>50%).

Variables	Adjusted Odds Ratio	95% Confidence Interval	p-value
Age	1.024	1.001–1.048	0.047
Diabetes mellitus	1.328	0.731–2.409	0.351
Hyperlipidaemia	1.335	0.732–2.433	0.346
Chronic kidney disease	1.175	0.534–2.591	0.689
Left ventricular mass index (g/m^2^)	0.975	0.965–0.985	<0.001
Lateral E:e’/EDV > 0.11 (ml^-1^)	3.030	1.684–7.148	<0.001

**Figure 2:**
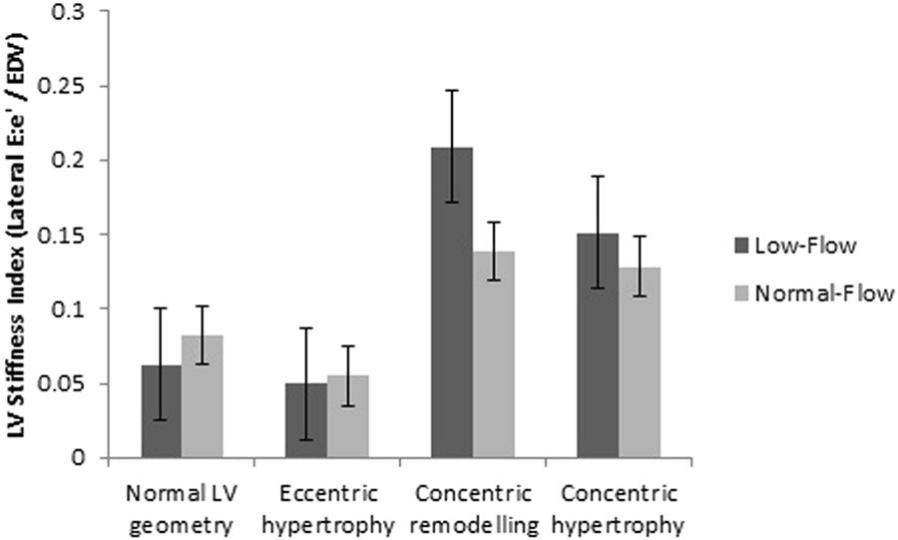
LV Stiffness according to flow-category and left ventricular geometry.

**Figure 3:**
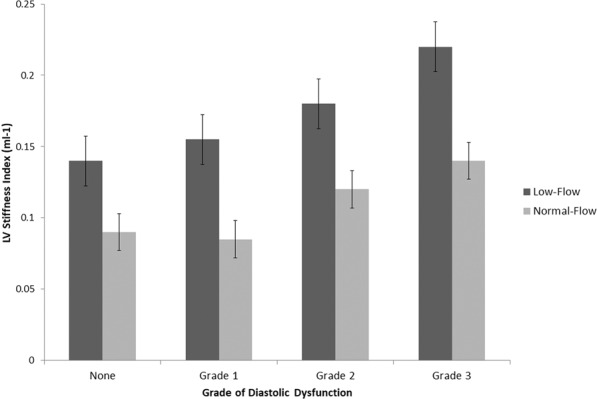
LV Stiffness index according to severity of diastolic dysfunction.

When comparing the various patterns of LV geometry, we found higher LV stiffness in patients with concentric remodelling and hypertrophy. This was especially noted in the patients with LF AS compared to their NF counterparts (Fig. 2). Furthermore, with increasing LV stiffness, we also demonstrated increasing severity of diastolic dysfunction (Fig. 3). This trend was statistically significant by Spearman’s rank correlation, and was more prominent in LF compared with NF AS. In fact, increased stiffness (>0.111 mL^−1^) was associated with older age, higher body mass index, lower left ventricular mass index and lower end-systolic wall stress. Prevalence of cardiovascular risk factors and comorbidities as well as degree of AS severity were similar (Table 3).

**Table 3:** Clinical and echocardiographic parameters associated with increased stiffness in severe AS.

Variables	High LV Stiffness Index >0.11 ml^-1^ (n = 166)	Low LV Stiffness Index ≤0.11 ml^-1^ (n = 184)	Mean Difference/Odds Ratio (95% Confidence Interval)	p-value
*Clinical Parameters*				
Age (years)	73.3 (±12.6)	70.2 (±13.0)	3.0 (0.34–5.74)	0.027
Body mass index (kg/m^2^)	24.0 (±4.8)	21.0 (±9.5)	2.9 (1.3–4.6)	<0.001
Hypertension	63.1%	65.0%	0.92 (0.57–1.50)	0.740
Diabetes mellitus	43.6%	35.0%	1.43 (0.89–2.31)	0.138
Hyperlipidaemia	43.0%	49.6%	0.76 (0.48–1.22)	0.258
Atrial fibrillation	9.4%	10.2%	0.91 (0.42–1.99)	0.823
Ischaemic heart disease	29.5%	31.4%	0.92 (0.55–1.52)	0.729
Chronic kidney disease	16.8%	10.9%	0.61 (0.31–1.21)	0.155
*Echocardiographic Parameters*				
Left ventricular ejection fraction (%)	67.0 (±7.5)	65.4 (±7.0)	1.6 (0.1–3.1)	0.041
Left ventricular mass index (g/m^2^)	114.7 (±36.6)	123.7 (±37.5)	−9.0 (−16.9–1.1)	0.026
End-systolic wall stress (x 10^3^ dyn/cm^2^)	57.3 (±16.3)	67.1 (±19.0)	−9.8 (−13.6–6.1)	<0.001
Aortic valve area (cm^2^)	0.78 (±0.23)	0.80 (±0.17)	−0.02 (−0.07–0.02)	0.243
Transaortic peak velocity (cm/s)	381.1 (±96.8)	370.8 (±97.1)	10.3 (−10.1–30.7)	0.322
Transaortic mean pressure gradient (mmHg)	36.3 (±20.8)	34.7 (±19.7)	1.6 (−2.6–5.9)	0.456

The values are expressed as mean (±standard deviation).

**Figure 4:**
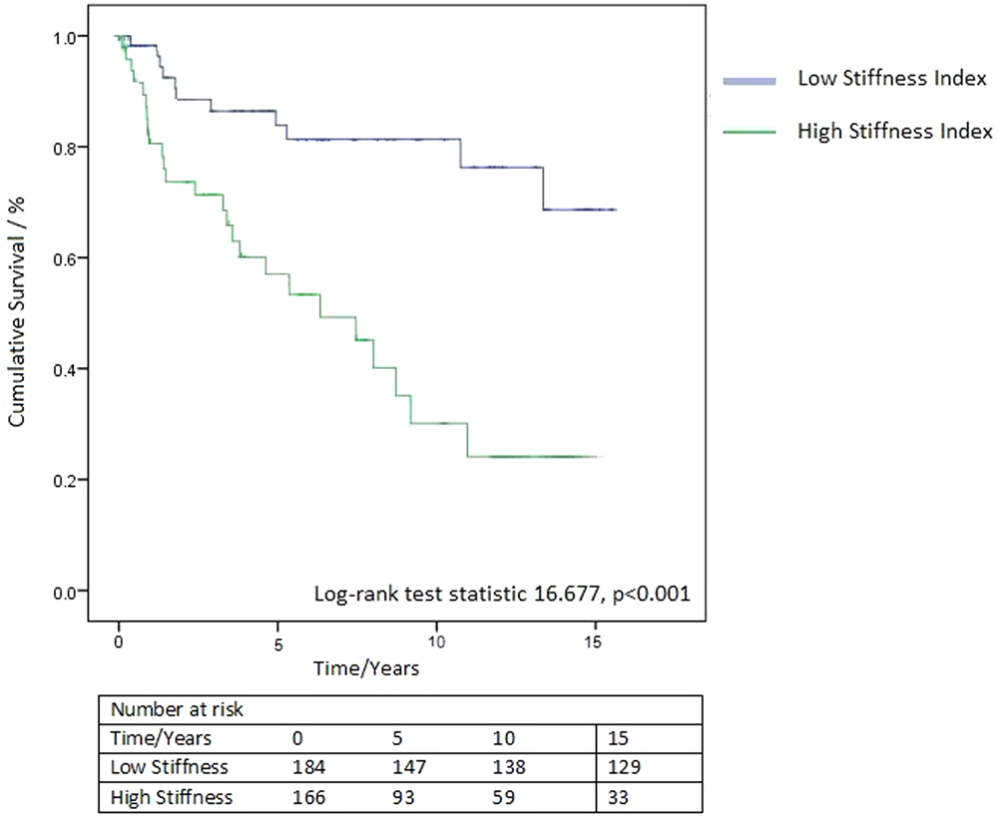
Increased mortality in patients with increased LV stiffness.

When comparing clinical outcomes of patients (average length of follow-up 6.2 ± 5.0 years) with high LV stiffness (>0.111 mL^−1^) compared with low stiffness, patients with increased LV stiffness had significantly higher mortality (42.9% vs 31.4%, Kaplan-Meier Log-rank test statistic 16.677, p < 0.001, Fig. 4). On multivariate Cox regression analysis, increased LV stiffness (>0.111 mL^−1^) was demonstrated to be independently associated with increased mortality (Hazard Ratio 2.283, 95% CI 1.318–3.968, p = 0.003) after adjusting for age, body mass index, LV mass index and cardiovascular risk factors such as diabetes mellitus, hypertension and hyperlipidaemia (Table 4).

**Table 4:** Multivariate Cox regression showing parameters associated with mortality on subsequent follow-up in patients with severe AS.

Variables	Hazard Ratio	95% Confidence interval	p-value
Lateral E:e’/EDV > 0.11 ml^-1^	2.283	1.318–3.968	0.003
Age (years)	1.031	1.009–1.054	0.007
Body mass index (kg/m^2^)	0.951	0.903–1.001	0.053
LV mass index (g/m^2^)	1.001	0.997–1.006	0.570
Hypertension	1.659	0.953–2.886	0.073
Diabetes mellitus	1.356	0.850–2.165	0.202
Hyperlipidaemia	2.001	1.236–3.236	0.005

## Discussion

To our knowledge, this was the first study to assess differences in LV stiffness between patients with normal-flow and low-flow severe AS. The main findings of this study were that (1) patients with LF AS had higher LV stiffness than those with normal-flow AS, (2) increasing LV stiffness is related to concentric remodeling and hypertrophy, as well as more advanced diastolic dysfunction; and (3) AS subjects with increased LV stiffness had higher mortality than those with lower LV stiffness.

Paradoxical LF AS has been shown to form a significant proportion (up to 30%) of Asian cohorts^13^. Patients with LF AS tended to be older and have increased cardiovascular risk factors such as diabetes mellitus and hyperlipidemia. Understanding this subgroup of patients would therefore be important in the management of AS.

This novel echocardiographic index to estimate LV stiffness has been evaluated and validated against the gold standard of cardiac catheterization with pressure-volume loop analysis^3^. This parameter indexed the tissue Doppler assessment of diastolic function against the measured end-diastolic volume as a surrogate measure of LV stiffness. It has previously been employed to study patients with heart failure with preserved ejection fraction (HFpEF)^2^. Though LV stiffness has not been validated in the setting of LV pressure overload, this study proposes that the findings and application may be extrapolated to the AS population. A published study has shown that *E/e’* ratio correlates with filling pressures in AS. The study demonstrated that *E/e’* ratio was found to be the best single Doppler predictor of elevated filling pressures in patients with severe AS. The *E/e’* ratio correlated significantly with LV pre-A pressures (r = 0.75, p < 0.001), and the LV end-diastolic pressure (r = 0.78, p < 0.001)^23^.

Indeed, for patients with AS, the high chronic afterload may lead to LV remodelling and fibrosis, resulting in increased stiffness and diastolic dysfunction. Increased LV stiffness from increased afterload had been previously shown in patients with essential hypertension as well as in HFpEF^24,25^. In HFpEF, increased LV stiffness has been shown to be associated with symptoms of dyspnea and reduced exercise tolerance, and correlated with disease progression^26–28^.

LV diastolic dysfunction has also been shown to be of increasing importance in severe AS. Prior studies had established the relationship between diastolic dysfunction and patients with severe AS with preserved LVEF^29^. LV diastolic dysfunction, and subsequently indices of LV myocardial and chamber stiffness had been associated with symptomatic AS, especially dyspnea^30^. These findings had suggested that heart failure symptoms in severe AS were driven by high LV filling pressures consequent to LV hypertrophy and diastolic dysfunction^31^. The actual relationship between LV remodeling and diastolic filling however is highly complex, where both early relaxation and passive LV filling later in diastole have demonstrated important roles in the development of heart failure symptoms^32^.

We therefore postulated that a similar phenomenon described in HFpEF may also be observed in our population of patients with LF compared to NF severe AS^33–35^. True enough, we found increased LV stiffness in patients with LF compared to NF severe AS. As LV stiffness appeared to be a prominent feature in patients with LF AS, it may also be related to the development of symptoms such as dyspnea and reduced effort tolerance. In the context of severe AS, the development of such symptoms would be a Class I indication for aortic valve replacement^36^. Classically, patients with severe AS who develop dyspnea would have a median survival of 2 years, although whether this holds true for patients with LF severe AS remained unclear^1,3^. By comparison, patients with NF tended to have lower LV stiffness, and the correlation between increasing LV stiffness and increasing degree of diastolic dysfunction was not demonstrated in the NF group.

In our study, we observed that increasing LV stiffness is predominantly related to concentric remodeling and hypertrophy. This trend was most prominent in patients with LF AS. Furthermore, increasing LV stiffness was also associated with increasing diastolic dysfunction in LF AS. We postulate that LV stiffness in LF AS may be secondary to chronic sympathetic stimulation of the beta-adrenoreceptor, associated with higher plasma concentrations of brain natriuretic peptide (BNP), as evidenced by significant LV remodelling and diastolic dysfunction^37,38^. This suggested that targeted therapy against LV remodeling in the form of beta-blockade (as used in heart failure) or renin-angiotensin blockade may be beneficial in LF AS to reduce harmful LV remodeling^39^.

The LV stiffness index also appeared to have prognostic value. Even after adjusting for age, body mass index, LV mass index and cardiovascular risk factors such as hypertension, hyperlipidaemia and diabetes, increased LV stiffness index remained independently associated with increased mortality. Regardless of flow-category, patients with higher stiffness showed significant higher mortality on subsequent follow-up. Other parameters conventionally used to predict outcomes in severe AS such as valvuloarterial impedance and systemic arterial impedance had been criticized to be highly flow-dependent^14,15^ and thus had a limited role in prognosis for patients with LF severe AS with preserved LVEF^15,16^. The LV stiffness index appeared to be more useful for prognosis in the LF subgroup.

We therefore found that an echocardiographic parameter of LV stiffness has the potential to guide prognostication and management of patients with LF severe AS. This index may be more useful compared to other more flow-dependent indices and predictors which may be less reliable in LF AS. The pathophysiological process in LF AS with preserved LVEF may parallel that of HFpEF and other disease processes where increasing LV stiffness and diastolic dysfunction results in morbidity and mortality.

## Limitations

This study was retrospective and examined a moderately-sized cohort of index echocardiographic studies for patients diagnosed with isolated severe AS. It was however prospective in terms of clinical outcomes. As this was a cross-sectional study, there may have been lead-time bias as subjects were studied at different time points of the natural progression of AS. There was also no follow-up echocardiographic data, which may be useful in evaluating the progression of AS severity over time. Specific biomarkers such as brain natriuretic peptide could have been compared to the LV stiffness estimation, however this was not investigated in the present study. In addition, we did not evaluate the prognostic significance of LV stiffness in surgical management of severe AS. Although we did not examine the role of medical therapy on LV stiffness, renin-angiotensin blockade had previously been described to be associated with a lower prevalence of pathological LV remodelling in severe AS^39^. Nevertheless, our findings remained hypothesis-generating, and the role of medical therapy in improving LV stiffness may be an important subject for future prospective studies with serial echocardiographic analyses.

## Conclusions

LV stiffness was demonstrated to be significantly higher in patients with LF AS, and this was related to the development of LV concentric remodelling, concentric hypertrophy and diastolic dysfunction. An increased LV stiffness index was associated with poorer mortality outcomes in medically-managed severe AS. This novel non-invasive echocardiographic estimation of LV stiffness may be an important prognostic tool in medically-managed AS.
